# Dengue Patients Exhibit Higher Levels of PrM and E Antibodies Than Their Asymptomatic Counterparts

**DOI:** 10.1155/2015/420867

**Published:** 2015-03-01

**Authors:** Adeline Syin Lian Yeo, Anusyah Rathakrishnan, Seok Mui Wang, Sasheela Ponnampalavanar, Rishya Manikam, Jameela Sathar, Santha Kumari Natkunam, Shamala Devi Sekaran

**Affiliations:** ^1^Department of Medical Microbiology, Faculty of Medicine, University of Malaya, Lembah Pantai, 50603 Kuala Lumpur, Malaysia; ^2^Institute for Medical Molecular Biotechnology, Universiti Teknologi MARA, Sungai Buloh Campus, Jalan Hospital, 47000 Sungai Buloh, Selangor, Malaysia; ^3^Division of Infectious Diseases, Department of Medicine, University Malaya Medical Centre, 50603 Kuala Lumpur, Malaysia; ^4^Department of Trauma and Emergency Medicine, University Malaya Medical Centre, Lembah Pantai, 50603 Kuala Lumpur, Malaysia; ^5^Hospital Ampang, Pandan Mewah, Jalan Mewah Utara, 68000 Ampang, Selangor, Malaysia; ^6^Hospital Tengku Ampuan Rahimah, Persiaran Tengku Ampuan Rahimah, 41200 Klang, Malaysia

## Abstract

Dengue virus infection is a common tropical disease which often occurs without being detected. These asymptomatic cases provide information in relation to the manifestation of immunological aspects. In this study, we developed an ELISA method to compare neutralizing effects of dengue prM and E antibodies between dengue patients and their asymptomatic household members. Recombinant D2 premembrane (prM) was constructed, cloned, and tested for antigenicity. The recombinant protein was purified and tested with controls by using an indirect ELISA method. Positive dengue serum samples with their asymptomatic pair were then carried out onto the developed ELISA. In addition, commercially available recombinant envelope (E) protein was used to develop an ELISA which was tested with the same set of serum samples in the prM ELISA. Asymptomatic individuals showed preexisting heterotypic neutralizing antibodies. The recombinant prM was antigenically reactive in the developed ELISA. Dengue patients had higher prM and E antibodies compared to their household members. Our study highlights the neutralizing antibodies levels with respect to dengue prM and E between dengue patients and asymptomatic individuals.

## 1. Introduction

Dengue virus (DENV) is a* Flavivirus* with four serotypes (DENV1–4). Approximately, 3.6 billion people who are about 55% of the world's population across the globe are at risk of being infected with dengue [1]. A recent annual report suggested that there are 390 million dengue infections that occur yearly of which about 96 million represent dengue fever (DF), dengue hemorrhagic fever (DHF), and dengue shock syndrome (DSS), whereas the other 300 million represent mild or asymptomatic cases [2]. Infection by a particular serotype confers a lifelong immunity against the homologous serotype but only limited cross-protection to the remaining three serotypes [3]. Primary DENV infections are often asymptomatic and will generate immunity to the homologous strain. However, about 90% cases of dengue with warning signs reportedly occur following second exposure to a heterologous strain of DENV [4]. The presence of cross-reactive, nonneutralizing antibodies generated during a primary infection has been suggested to enhance the pathogenicity of subsequent infections via the process of antibody-dependent enhancement (ADE) [5]. The occurrence of ADE could substantially increase the risk of manifesting severe dengue during subsequent infections especially in the asymptomatic cohort. Therefore, asymptomatic dengue cases should not be taken lightly as they provide ample opportunities for researchers to explore the host immune factors.

The significance of the prM protein is undeniably important as this structural protein plays an important role in viral infectivity. During viral infection, dengue virions are assembled on the membrane of the endoplasmic reticulum (ER) and the virus buds in the lumen of the ER as immature virions. The immature virion particles will undergo transition to mature particles during secretion out of the infected cells [6]. As the prM protein is the precursor for the formation of M protein, cellular protease cleaves prM protein to generate the mature M protein in the trans-Golgi compartment. The process of intracellular DENV maturation appears to be inefficient because many immature and partially mature virions are also released from the infected cells [7, 8]. Moreover, recent studies have shown that partially mature and even fully immature particles can be infectious under certain conditions [9, 10]. While generally the host anti-DENV response is dominated antibodies that target the envelope (E) protein, recently an immunological study showed that prM-specific antibodies were also dominant in both primary and secondary infections [9]. The prM-specific antibodies were observed to be highly cross-reactive and nonneutralizing. When complexed with immature DENV, it has the ability to render normally noninfectious immature DENV highly infectious [10]. Like mentioned earlier, the major immunogen for inducing neutralizing antibodies is the E protein; however, these antibodies show cross-reactivity with other DENV serotypes [11]. The glycosylated E protein of the DENV is known to be one of the most important proteins for neutralization due to its role in virus attachment to cells and fusion with membranes. Neutralizing antibodies directed towards the E protein appear to be pivotal antibody that mediates homologous protection against reinfection. Antibodies against E have been shown to inhibit viral binding to cells and to neutralize viral infectivity* in vitro* [11]. Serum antibodies against DENV E protein have been the focus of several studies as this is the main antigen on the virion surface and the target of neutralizing antibody. Thus, this gives the opportunity to study the prM and E protein with regard to its nature as a target for neutralizing antibody. Here, we sought to develop an ELISA to detect prM and E antibodies and to compare the levels of these antibodies in dengue patients and their accompanying asymptomatic household member.

## 2. Materials and Methods

### 2.1. Sample Collection

Blood samples were collected from Hospital Tengku Ampuan Rahimah (Klang), Hospital Ampang, and University Malaya Medical Centre (UMMC). The study protocols were approved by the institutional review board of the University of Malaya Medical Centre (Ethics number 782.90) and from both Ampang and Klang Hospitals (Ethics no. NMRR-10-683-6420). Written informed consent from patients and asymptomatic donors were obtained prior to blood collection, and the study was conducted in accordance with the Declaration of Helsinki. The blood collection was carried out on two groups in the infectious diseases ward: (1) patients suspected to have dengue according to the WHO dengue classification and (2) their accompanying household members. Serum samples were collected for the acute, defervescence, and the convalescence stage of dengue patient; however, only one point of blood sampling was done for the suspected asymptomatic household member. This is because the presumably healthy individuals whose cognate household members were dengue positive were recruited on a voluntary basis for the study.

### 2.2. Dengue Diagnostic and Confirmatory Tests

All serum samples collected were subjected to dengue diagnostic tests as previously carried out: real-time RT-PCR [12],* in-house* IgM capture ELISA [13], DENV antigen via NS1 assay (Pan-E dengue early ELISA kit; Panbio, Australia), and hemagglutination inhibition (HI) test [14]. Positive dengue samples were then subjected to plaque reduction neutralization test (PRNT) [15].

### 2.3. Construction, Cloning, and Expression of Recombinant prM Protein

Dengue virus type 2, strain New Guinea C (prototype strain), was obtained from the Department of Medical Microbiology, University of Malaya. DENV supernatant from virus stock was subjected to RNA extraction by using High Pure Viral RNA Isolation Kit (Roche Diagnostics) according to the manufacturer's protocol. Primers flanking the DENV prM region were designed with specific oligonucleotide containing restriction enzyme sequences: forward primer: ACAGTGGATCCGTTCCATTTAACCACACGT and reverse primer: TATAAGCTTCTATGTCATTGAAGGAGCGAC. The RT-PCR amplification of the target gene was then carried out and the PCR products were then analysed by gel electrophoresis. The amplified PCR products for DENV2 obtained from RT-PCR consisted of the appropriate restriction sites engineered into the primer sequences. The PCR products and pET28b(+) vector (Novagen pET System) were then digested and ligated and cloning was performed in DH5*α* (Novagen) competent cells through heatshock transformation. Positive transformants were selected on LB plate supplemented with kanamycin antibiotic. The positive clones were checked for the correct sequence and orientation by restriction enzyme digestion and by sequencing. Plasmid carrying the prM insert was extracted and then transformed into expression host* E. coli* BL21 (DE3) for protein expression.

### 2.4. Testing for Protein Expression

Expression of target protein was tested with SDS-PAGE and western blotting. The presence of fusion protein was detected with 6X His-Tag HRP antibody. For total protein analysis, the bacterial pellet was resuspended in 1X nonreducing sample buffer. The cell lysate was then heated at 100°C for 10 minutes and spun at 13000 rpm for 10 minutes before loading onto SDS-PAGE. To check the solubility of the target protein, the bacterial pellet from a 10 mL culture was washed once with 1X PBS and resuspended in 1 mL of inclusion body buffer (IBB) (50 mM Tris-HCl pH 8, 1 mM EDTA, and 100 mM NaCl). The cell suspension was sonicated for 3 times of 1 minute each at 4°C. The sonicated lysate was then spun at 12000 g for 15 minutes at 4°C. The extract was collected and the pellet was resuspended in 0.5 mL of IBB. SDS-PAGE analysis was performed and the protein bands were stained with Coomassie blue and then transferred to a nitrocellulose membrane by electrotransfer in Towbin's transfer buffer in the western blot assay.

### 2.5. Scale-Up, Purification, and Quantitation

The method used for purification of protein with His-Tag system was adapted from the pET system manual (Novagen, Madison, WI, USA). The stocks of 8X charged buffer (400 mM NiSO_4_), 8X binding buffer (40 mM imidazole, 4 M NaCl, and 160 mM Tris-HCl; pH 7.9), and 4X elute buffer (4 M imidazole, 2 M NaCl, and 80 mM Tris-HCl; pH 7.9) were diluted to 1X with sterile deionized water before use. His-Bind resin was mixed by gentle inversion and was added to a column using a wide-mouth pipette. The resin was allowed to pack under gravity flow (2 mL of settled resin). The column was washed with 7.5 mL sterile deionized water charged with 12.5 mL 1X charged buffer, followed by equilibration with 7.5 mL 1X binding buffer. The cells from a 200 mL induced culture were harvested by centrifugation at 5000 g at 4°C for 5 minutes. The supernatant was decanted and the cell pellet was resuspended in 20 mL of ice-cold 1X binding buffer. The cells were vortexed vigorously and the DNA was sheared using a sonicator at full power for 10 times of 1 minute each at 4°C. The cell lysates were then centrifuged at 39,000 g for 20 minutes at 4°C. The supernatant was transferred to a fresh tube and was filtered through a 0.2 *μ*m membrane before being loaded onto column. The binding buffer was allowed to drain to the top of the column bed before loading the column with the prepared extract. The column was then washed with 25 mL of 1X binding buffer, followed by 15 mL of 1X washing buffer. The protein was eluted in 15 mL of 1X elute buffer. The protein concentration was determined with UV spectrophotometer at 260 nm and 280 nm using the Warburg/Christian method with a semiquartz-glass cuvette.

### 2.6. Development of IgG Indirect ELISA

An indirect ELISA was developed to detect prM and E antibodies. The recombinant E protein was obtained commercially (Fischer Antibodies) while the recombinant prM antibody was prepared as described above. Optimization was carried out under different conditions through the chessboard/checkerboard titrations (CBTs). The variables were the percentage of blocking buffer, the concentration of the recombinant proteins used, the dilution of the antibody detecting the antigens (prM and E), and the secondary antibody conjugated with HRP. Optimization of both E and prM ELISA was conducted simultaneously.

### 2.7. Testing of Recombinant Proteins with Samples

Clinical samples of the dengue patients and their accompanying household member's sera were then tested. Briefly, 96-well microtiter plates were coated with either the purified prM or E proteins, diluted to a final concentration of 10 *μ*g/mL in phosphate-buffered saline (PBS). The plate was covered with adhesive plastic and incubated in 4°C overnight. The coating solution was removed and the plate was washed six times by filling the wells with wash buffer (both areas) (PBS-0.05% Tween-20). The solution was removed and casein (20% milk in PBS) was then dispensed into the coated wells to block the remaining protein-binding sites. The plate was then covered with adhesive plastic and incubated for at least 2 hours at 37°C. The plate was then washed twice with the washing solution. Clinical serum samples with known titers were then added into the wells and incubated for 1 hour at 37°C. The plate was then washed with washing solution and a 100 *μ*L of IgG-HRP was added into each well and was allowed to incubate for 1 hour at 37°C. The plate was then washed 6 times with the washing solution followed by addition of 100 *μ*L of substrate solution. Lastly, 50 *μ*L of stop solution was then added into the wells and absorbance of each of the well was read with a plate reader at 490 nm.

## 3. Results

### 3.1. Sample Collection and Dengue Confirmatory Tests

The numbers of blood samples collected from the three hospitals were 448 patients and 62 household members. Samples were considered as dengue positive if (1) DENV nucleic acid was detected; (2) DENV NS1 antigen was present; or (3) (i) DENV IgM seroconversion occurred in paired sera, (iii) dengue total antibodies had a fourfold rise in titers in paired sera, or (iv) a combination of the above. Patients who were either IgM positive or with HI titer of above 1280 but without seroconversion or fourfold rise were considered presumptive. A total of 270 patients and 3 household members were confirmed to be dengue positive while 81 patients and 14 household members were presumptive dengue.

In this study, 58 dengue patients and 62 household members were included in forthcoming analyses (whereby 4 dengue patients had more than 1 household member). A total of 7 patients and 1 household member were RT-PCR positive. Serotyping revealed that 4 patients had DENV-1 and 3 patients were DENV-3 positive, while the asymptomatic household members had a DENV-3 infection (Table 1). The in-house IgM-Capture ELISA was performed by calculating the positive/negative (P/N) ratio. Samples tested for IgM against DENV was considered positive if the P/N ratio was ≥2.0. A P/N ratio from 5 to 8 was considered highly positive while the P/N ratio of low positive sample was between 2 and 4. DENV IgM antibodies were detected in 35 (60.3%) patients' acute-phase serum samples while, in the convalescent-phase samples collected, 28 samples (66.7%) were positive. Meanwhile, in the asymptomatic group, 16 samples (25.8%) of the patients' household members showed positive results in IgM-captured ELISA. Hemagglutination inhibition (HI) assay was carried out on all serum samples to detect the total antibodies in the subjects and also to differentiate the samples to primary and secondary infection. The peak titers below 1 : 1280 in acute-phase serum samples indicate a primary infection and the peak titers more than 1 : 1280 in acute-phase serum samples indicate a possible secondary infection. A recent infection was determined with 4-fold or even greater increase in titers between acute and convalescent serum samples [16]. Among the acute-phase serum samples from the patients, 29 (50.0%) show peak titers at or below 1 : 1280, while 15 (25.9%) of the patients' acute-phase serum samples show titers exceeding 1 : 1280. This suggests that half of the patients were having primary DENV infection, whereas the other 25.9% of the patients were having secondary DENV infection. We also observed that 14 (24.1%) of the patients' acute-phase sera and 8 (19.0%) of the patients' convalescent-phase sera show HI titer below 1 : 10. There were 19 patients (45.2%) having 4-fold rise in the titers between acute and convalescent serum samples, indicating that these patients are having a recent infection. In the asymptomatic group, 3 samples (4.8%) were having HI titers more than 1 : 1280, 38 (61.3%) were having HI titers below 1 : 1280, and 21 samples (33.9%) from the asymptomatic group show HI titers of <10 in their serum samples.

### 3.2. Plaque Reduction Neutralization Tests (PRNTs)

Plaque reduction neutralization tests were done to verify and determine the serotype specific neutralizing antibodies in the serum samples collected. Thirty-five samples of complete paired acute and convalescent sera which were dengue positive, together with their accompanying household members, were tested for PRNT.  Table 2 shows the comparison of neutralizing antibodies between dengue patients and their asymptomatic household members. In both the dengue patients and the asymptomatic household members, the neutralizing antibodies are detected in monotypic (only one serotype) and heterotypic (on more than one serotype) infection [16]. It was observed that monotypic neutralizing antibodies are detected more frequently in both the patients and their household members with 48.6% (acute sera) and 28.6%, respectively. However, the number of heterotypic neutralizing antibodies increased in acute sera to convalescent sera with more than half of the convalescent sera having heterotypic neutralizing antibodies. Asymptomatic household members have the highest percentage at 45.7% having no neutralizing antibodies towards DENV infection.

### 3.3. Expression of Recombinant prM Protein

**Figure 1:**
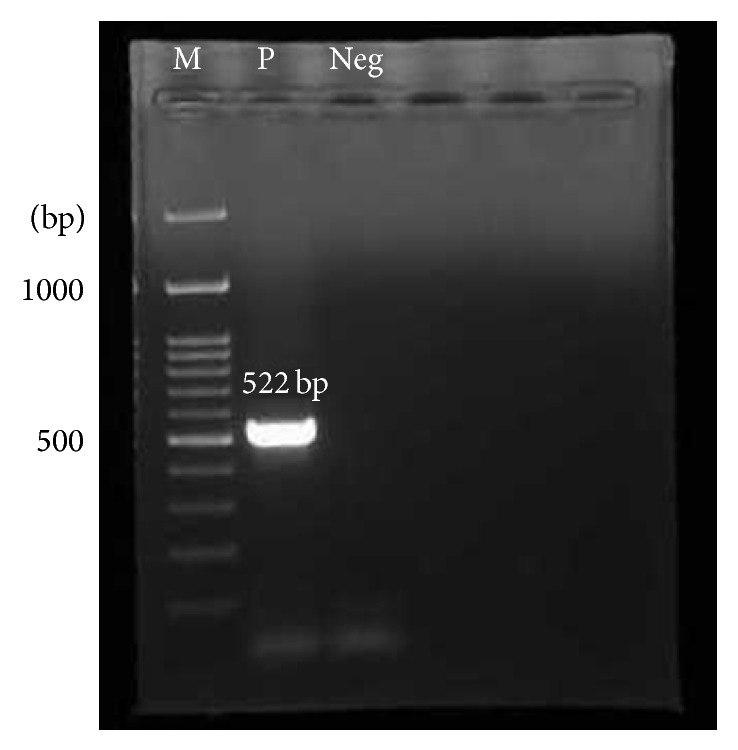
PCR product flanked with restriction enzyme sites. The expected prM plus restriction enzyme sites PCR product size was 522 bp. Lane M: 100 bp DNA ladder marker (Fermentas); Lane P: DENV2 prM + restriction enzyme site PCR product; Lane NEG: negative control.

**Figure 2:**
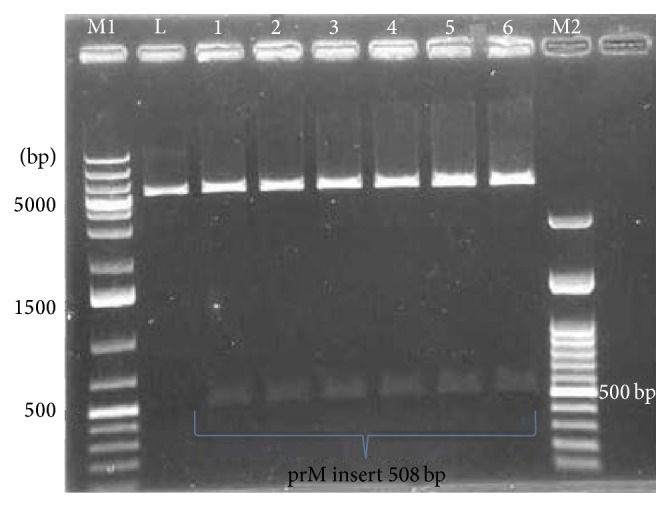
Restriction enzyme digestion profile of the recombinant plasmid DNA. Digestion of the recombinant clone produced a vector band and prM insert band at 508 bp. Lane M1: GeneRuler 1 kb plus DNA ladder marker (Fermentas); Lane L: linearized pET28b(+) plasmid with* Bam*HI digestion with expected size 5368 bp; Lanes 1–6 are positive clones selected randomly out of 42 positive clones obtained. Shown here are the vectors at 5343 bp and the prM insert at 508 bp upon* Hind*III and* Bam*HI digestion. Lane M2: 100 bp DNA ladder marker (Fermentas).

**Figure 3:**
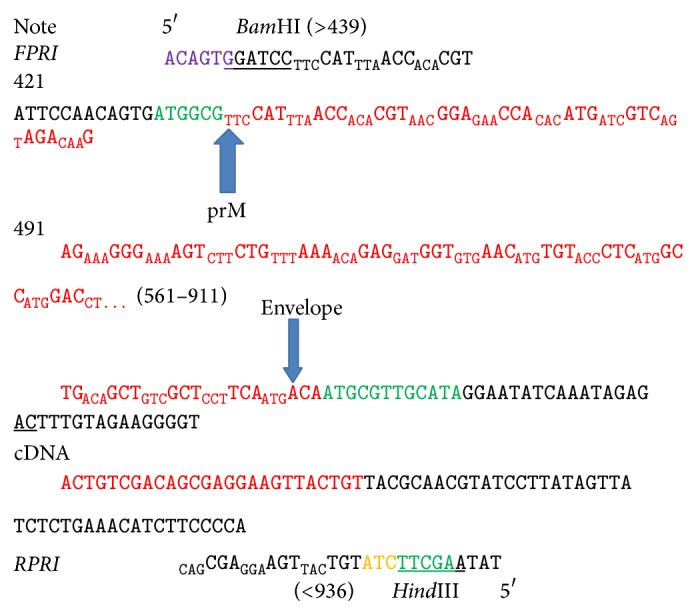
Sequence analysis of the recombinant prM plasmid. Plasmid was flanked with restriction enzymes* Bam*HI and* Hind*III at 5′ and 3′ of the target prM gene.

The RT-PCR amplified DENV2 prM region was used for the construction of recombinant plasmid DNA (Figure 1). The recombinant plasmid DNA constructed was screened for prM insert by restriction enzyme digestion to identify plasmids containing the correct insert and Figure 2 represents DENV prM gene cloned into pET28b(+) vector. The gel obtained showed that the digestion of the vector with restriction enzymes was able to separate the target prM protein at its correct size. The sequence, orientation, and reading frame were further determined and confirmed by sequencing analysis shown in Figure 3.

**Figure 4:**
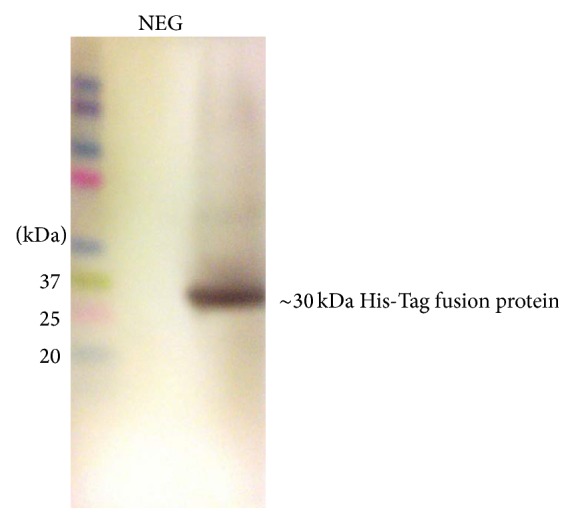
Western blot to detect the presence of His-Tag fusion protein. The His-Tag was detected with the His-Tag antibody at ~30 kDa while the negative control is empty pET28b(+) without target prM gene.

**Figure 5:**
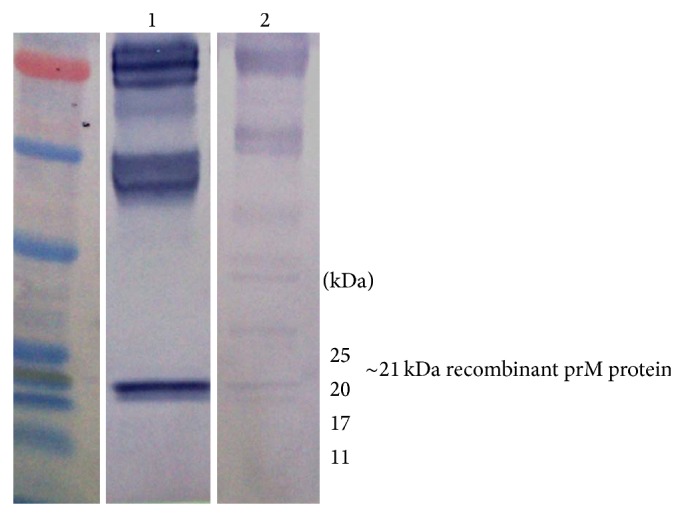
Protein antigenicity with polyclonal positive dengue IgG serum. Strip 1: positive control of DENV and Strip 2: recombinant prM protein detected at ~21 kDa.

Expression of target protein was checked by Coomassie blue staining of SDS-PAGE and immunoblotting. In Figure 4, the presence of His-Tag fusion protein was detected by anti-HisG antibody which recognizes the sequence -His-His-His-His-His-His-Gly (6 × His-Gly epitope) on the fusion protein while the antigenicity of the expressed protein was further detected by using a polyclonal positive dengue IgG serum in Figure 5 which detects the prM target protein.

Based on the western blot results obtained, the DENV prM gene was successfully expressed as a pET28b recombinant protein in* E. coli* and the expressed product were reactive with serum. After purification, the final concentration of the purified prM recombinant protein was 200 *μ*g/mL, which was then used to develop an ELISA system.

### 3.4. Development of Recombinant ELISA

After obtaining the recombinant protein, the ELISA was first developed by optimization through the CBT method. The optimized concentrations for the recombinant prM and E proteins were 5 *μ*g/mL while the most suitable dilution factor for the sera to be tested is 1 : 100. Meanwhile, the secondary conjugate with HRP was optimized to a dilution factor of 1 : 10000. In order to obtain the optimum concentration of antibody towards the developed ELISA, serum IgG of confirmed dengue with HI titre of >10240 was used as positive control while the negative control used was from non-dengue serum with HI titre <10.

### 3.5. Analysis of prM and E Antibodies with Developed ELISA

**Figure 6:**
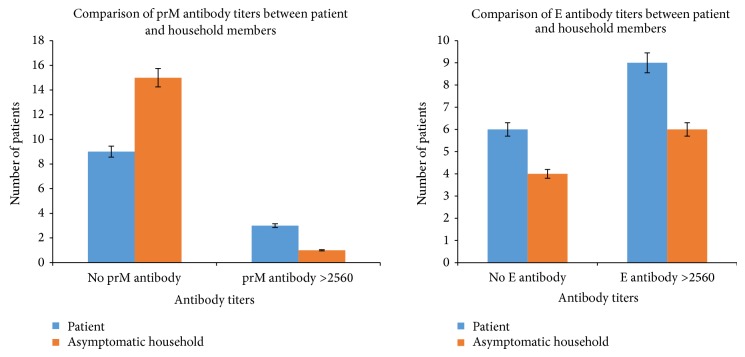
Comparison of prM and E antibody titers between dengue patients and their asymptomatic household members. *y*-axis represents the number of subjects. No prM/E antibody signifies antibody titers of <10 while antibody titers of >2560 signifies high antibody titers.

All samples that were tested for the PRNT were tested on the developed prM and E ELISA. As shown in Table 3, out of the 35 pairs, 31 samples were positive for either DENV prM or E or both with a P/N ratio of ≥2.0. The significant differences between the positive/negative (P/N) ratio of the ELISA optical density (OD) reading was within 2 standard deviation. These samples were titrated by ELISA to determine the levels of antibodies towards the prM and E antigen. From the analysis of antibody positivity towards the prM and E antigens between the dengue patients and their asymptomatic household members, it was observed that dengue patients showed higher percentage of positivity in prM antibodies at 74% (26 patients) compared to their asymptomatic household members at 57% (20 people). On the other hand, the asymptomatic household members showed higher positivity towards the E antigen at 89% (31 people) rather than the dengue patients who had 83% (29 patients) positivity towards the E antigen. Besides that, we observed that prM antibody titers were very low in the asymptomatic household members with most cases having titers of 2560 and below (≤2560). Comparison of antibody titers between prM and E in patients and asymptomatic household members is shown in Figure 6.

## 4. Discussion

The determinants of asymptomatic versus symptomatic outcome in DENV infections remain vaguely unexplored. Moreover, asymptomatic infections are not detected in routine surveillance and can only be captured in the context of prospective cohort or index cluster studies [17]. Hence, this study was focused on investigating the neutralizing antibodies levels among asymptomatic individuals in comparison with their symptomatic counterparts. We observed a large number of asymptomatic individuals showing high titers of antibody levels despite lacking clinical manifestations. More than half of the asymptomatic population had neutralizing antibodies towards DENV infection. Analysis of infection outcome is complicated by immune responses to multiple infections with different DENV serotypes, which can be either protective or pathogenic. In a cohort study in Thailand, circulating DENV serotype and the number of circulating serotypes were identified as factors influencing asymptomatic versus symptomatic infection outcome [18, 19]. Our observation shows a larger occurrence of homologous DENV infections as observed by the higher percentage of monotypic neutralizing antibodies among the asymptomatic individuals. This observation may be due to the existing antibody levels that persist in the asymptomatic individuals which confers protection towards DENV infection. Early experimental studies in DENV-naïve healthy volunteers also showed that infection with one DENV serotype confers immunity to that particular serotype for up to 18 months [20]. In fact, this protection is thought to be life-long. On the other hand, infection with one serotype only conferred short-term (<2 months) complete protection against heterologous infection with a different serotype [20].

Diagnostic assays of dengue infection were carried out in this study for case confirmation and differential diagnosis with other infectious diseases. The selection criteria for asymptomatic household members were solely based on the presumptive positive IgM levels and HI titers of a single sample because obtaining a second blood sample from this cohort was difficult because the collection was on voluntary basis. RT-PCR that was carried out was able to detect and serotype DENV and this usually occurs during first few days after onset of fever whereby a positive result indicates a current dengue infection. Because antibodies are only detected later, RT-PCR has become a primary tool to detect virus early in the course of illness [16]. According to WHO, blood sample is taken from a patient 5 or more days after the onset of symptoms, and laboratory diagnosis is best made using a test for IgM antibody to DENV [21]. Besides, we also determined the neutralizing antibodies titers by using the PRNT. PRNT is one of the gold standard tests used widely in the determination and quantification of neutralizing antibodies against DENV infection [22]. We showed that asymptomatic individuals had high presence of neutralizing antibodies against DENV infection. Despite having heterotypic infections, half of these asymptomatic individuals are protected against clinical dengue. Interestingly, they do not show any clinical manifestations albeit having the infection. One of the most likely explanations is that neutralizing antibodies may play an important role towards combating the disease. It has been reported that antibodies could play a greater role than immune cells in heterologous DENV infection [23].

Our study suggests that circulating neutralizing antibodies in the asymptomatic individuals may neutralize dengue virus infection. Preexisting neutralizing antibodies present in the asymptomatic individuals may function to eliminate the DENV infection. This is supported in recent data that antibodies against* Flavivirus* (West Nile virus and DENV) increase their neutralization capacity when left to interact for longer periods of time [24]. This suggests that even antibodies that bind poorly to virus particles will lead to inactivation of the virion if left to interact for long enough,* in vitro*. The DENV neutralization requires sufficient levels of neutralizing antibodies. The number of antibodies bound to a single virion to exceed the threshold of enhancement will depend on antibody avidity and the accessibility of epitopes on the virus particle [25]. In an antibody mediated neutralization study, the avidity of anti-*Flavivirus* monoclonal antibodies (mAbs) was shown to positively correlate with neutralization activity* in vitro* [26]. Conversely, antigenic variants may also reduce the strength of antibody binding rendering less sensitivity to neutralization. Studies have shown that genotypic variation of DENV reduces the neutralizing activity of antibodies against heterologous strains of the same DENV serotype which can be explained by differences in antibody affinity [27, 28].

Bacterial expression is usually one of the first choices in protein expression studies due to its simplicity and large yield of recombinant proteins [29]. However, information on expressing* Flavivirus* prM proteins using bacterial expression systems is scarce. Previously, there are known protein expressions that were done on the proteins E [30] and NS1 [31]. Besides, studies on the antigenic structure of DENV1 envelope and NS1 proteins in the form of recombinant fusion proteins expressed in* E. coli* [32] were also done. Hence, recombinant E protein was easily obtained commercially to expedite the study. All* Flavivirus* strains share common epitopes on the E protein which is the major antigen inducing neutralizing and protective antibodies [33]. Thus, the earliest immune response to a* Flavivirus* infection would be against the E, which is the major surface protein of the virus. In our study, we used prM protein as an antigen to develop a method to detect anti-prM antibodies in dengue virus infection. This is because recent studies have shown that anti-prM antibody is a major component of the serological response to DENV infection [9, 34].

Much interest has been put on the impact of structural dynamics of dengue virus. This is because the viral conformational structure determines the accessibility of epitopes which then determines the neutralization of the virus. In our current study, results showed that dengue patients elucidated more prM antibodies compared to their asymptomatic household members. Since prM is present in the immature virions, a host immune response to prM suggests that an infection has been established. This is because the maturation step of prM is often incomplete, resulting in a significant fraction of only partially mature virions released from infected cells [8]. These virions are infectious and have been shown to display both mature- and immature-like patches on the same virion [35], displaying antigenic characteristics of both types [36]. Interestingly, studies have shown that antibodies against prM have been shown to enhance wild type DENV infection [37] and the levels of prM antibodies were found to be higher in patients with secondary infections compared with sera from primary DENV infections [38].

In the present study, it was observed that dengue patients had higher prM than E antibody titers compared to their asymptomatic household members. The prM protein contain antigenic domain that may cross-react with the E antibody. There were cross-competition experiments that revealed this phenomenon, whereby it was shown that monoclonal antibodies (mAbs) generated recognize the same or overlapping epitopes instead of binding to distinct epitopes [39]. Antibodies supposedly bound to the E-prM protein surprisingly recognized the E protein as well. Therefore, the cross-reactivity between the epitopes of these structural proteins should further be investigated. The reactivity of mAbs with both prM and E suggests a quaternary epitope with shared sites on the heterodimeric prM-E protein which in similar findings has been reported for mouse antibodies [40, 41]. Thus, consistent with a recent study [9], the prM antibodies may be cross-reactive among the DENV serotypes and may not be neutralizing but potently promote ADE.

## 5. Conclusions

In conclusion, serological testing of dengue virus infection among the asymptomatic individuals provides insights into the association of disease severity to the antibody levels that may render useful in future studies. The use of prM protein in diagnosing dengue virus infection should further be assessed in light of the extensive cross-reactions of antibodies to the binding on shared epitopes.

## Figures and Tables

**Table 1:** Summary of dengue diagnostic results.

	RT-PCR				IgM P/N ratio		Hemagglutination test		
	DENV1	DENV2	DENV3	DENV4	Positive	Negative	<1280	>1280	<10
Dengue patient									
Acute	4	0	3	0	35	23	29	15	14
%					60.3	39.7	50	25.9	24.1
Convalescence	N/A	N/A	N/A	N/A	28	14	14	20	8
%					66.7	33.3	33.3	47.6	19
Asymptomatic household %	0	0	1	0	16	46	38	3	21
					25.8	74.2	61.3	4.8	33.9

Total	4	0	4	0	79	83	81	38	43
					48.8	51.2	50.0	23.5	26.5

**Table 2:** Comparison of neutralizing antibodies detected in patients and their asymptomatic household members.

Category	Monotypic infection	Heterotypic infection	No neutralizing antibody detected	Total
	(%)	(%)	(%)	
Acute-phase patient sera	17	16	2	35
	(48.6)	(45.7)	(5.7)	

Convalescent-phase patient sera	15	19	1	35
	(42.9)	(54.3)	(2.8)	

Asymptomatic household members	10	9	16	35
	(28.6)	(25.7)	(45.7)	

Total	**42**	**44**	**19**	**105**
	**(40.0)**	**(42.0)**	**(18.0)**	

**Table 3:** Determination of antibody titers towards the prM and E antigen on the developed ELISA.

Patient			Asymptomatic household		
Identification	prM	Envelope	Identification	prM	Envelope
AH023	640	2560	AH023/F1	20	160
AH023	640	2560	AH023/F2	1280	5120
AH071	160	2560	AH071/F1	40	160
AH078	20	640	AH078/F1	*Negative *	640
AH090	*Negative *	*Negative *	AH090/F1	*Negative *	*Negative *
AH100	*Negative *	*Negative *	AH100/F1	*Negative *	*Negative *
AH101	320	160	AH101/F1	640	640
AH103	*Negative *	160	AH103/F1	*Negative *	640
AH147	320	640	AH147/F1	320	640
AH148	1280	160	AH148/F1	*Negative *	40
AH155	5120	>10240	AH155/F1	*Negative *	640
AH155	*Negative *	*Negative *	AH155/F2	*Negative *	*Negative *
AH166	2560	>10240	AH166/F1	160	640
AH192	*Negative *	640	AH192/F1	10	640
AH226	*Negative *	*Negative *	AH226/F1	*Negative *	10
AH232	20	640	AH232/F1	20	2560
AH232	20	640	AH232/F2	*Negative *	20
AH240	160	640	AH240/F1	160	160
KH015	2560	20	KH015/F1	10	160
KH049	*Negative *	*Negative *	KH049/F1	160	>10240
KH076	20	320	KH076/F1	20	20
KH114	640	2560	KH114/F1	*Negative *	1280
KH121	640	20	KH121/F1	*Negative *	1280
KH125	160	640	KH125/F1	20	640
KH144	1280	5120	KH144/F1	*Negative *	40
KH145	20	640	KH145/F1	320	1280
KH148	160	160	KH148/F1	80	160
KH149	160	640	KH149/F1	80	80
KH157	1280	1280	KH157/F1	320	640
KH162	*Negative *	2560	KH162/F1	640	2560
KH165	640	640	KH165/F1	*Negative *	160
KH166	10	160	KH166/F1	*Negative *	160
KH168	320	640	KH168/F1	640	5120
KH204	1280	2560	KH204/F1	2560	>10240
KH217	*Negative *	*Negative *	KH217/F1	*Negative *	*Negative *
